# Syntax processing properties of generic cortical circuits

**DOI:** 10.1186/1471-2202-14-S1-P283

**Published:** 2013-07-08

**Authors:** Renato Duarte, Peggy Seriès, Abigail Morrison

**Affiliations:** 1 Bernstein Center Freiburg, Albert-Ludwig University of Freiburg, Freiburg im Breisgau, 79104, Germany; 2 Institute for Adaptive and Neural Computation, School of Informatics, University of Edinburgh, Edinburgh, EH8 9AB, UK; 3 Institute of Neuroscience and Medicine (INM-6), Computational and Systems Neuroscience, Jülich Research Center, Jülich, 52425, Germany; 4 Institute of Cognitive Neuroscience, Faculty of Psychology, Ruhr-University Bochum, Bochum, 44801, Germany

## 

Higher cognitive functioning is assumed to be largely symbolic/representational and compositional in nature. At various processing stages, from perceptual to motor, discrete structural elements with intricate temporal dependencies are combined into increasingly complex constructs [1]. Mapping such complex computational processes to the underlying neuronal infrastructure and assessing the properties of the neuronal system responsible for their implementation is not straightforward, but it is likely to yield important insights into the nature of neural computation.

In order to address these issues, we adopt ideas and formalisms developed by theoretical linguistics to study the nature of rule-like or compositional behavior in the language domain, namely the acquisition of formal (artificial) grammars. The Artificial Grammar Learning (AGL) paradigm has a long tradition in psycholinguistic research (see, e.g. [2] for an overview), as a means to study the nature of syntactic processing and implicit sequence learning.

With mere exposure and without performance feedback, human beings implicitly acquire knowledge about the structural regularities implemented by complex rule systems.

In this work, we investigate to which extent generic cortical microcircuits can support formally explicit symbolic computations, instantiated by the same grammars used in the human AGL literature and implementing various types of local and non-adjacent dependencies between the sequence elements, thus requiring varying degrees of computational complexity and online processing memory to be adequately learned. We use concrete implementations of input-driven recurrent networks composed of noisy, spiking neurons, built according to the reservoir computing framework and dynamically shaped by a variety of synaptic and intrinsic plasticity mechanisms operating concomitantly [3]. Additionally, we compare supervised and unsupervised learning rules for the decoding algorithms, with varying degrees of biological plausibility. We show that, when shaped by plasticity, these models are capable of acquiring the structure of simple (regular) grammars. When asked to judge string legality (in a manner similar to human subjects), the networks perform at a qualitatively comparable level. We uncover which plasticity mechanisms are crucial for the task, with the aim of specifying a minimal model. Furthermore, the capability of the networks to process (bounded) recursive constructions including multiple patterns of non-adjacent dependencies accurately reflects recent results of human performance, highlighting inherent limitations imposed by the nature of neuronal processing.

## References

[B1] Battaglia FP Borensztajn G Bod R Structured cognition and neural systems: from rats to language Neuroscience and biobehavioral reviews 2012 36 1626 1639 10.1016/j.neubiorev.2012.04.004 22537592

[B2] Petersson KM Hagoort P The neurobiology of syntax: beyond string sets Philosophical transactions of the Royal Society of London Series B, Biological sciences 2012 367 1971 1983 10.1098/rstb.2012.0101 22688633 PMC3367693

[B3] Zheng P Dimitrakakis C Triesch J Network self-organization explains the statistics and dynamics of synaptic connection strengths in cortex PLoS computational biology 2013 9 1 e1002848 10.1371/journal.pcbi.1002848 23300431 PMC3536614

